# It is unclear how important CRISPR-Cas systems are for protecting natural populations of bacteria against infections by mobile genetic elements

**DOI:** 10.1073/pnas.1915966117

**Published:** 2020-10-29

**Authors:** Edze R. Westra, Bruce R. Levin

**Affiliations:** ^a^Environment and Sustainability Institute, Biosciences, University of Exeter, Penryn, TR10 9FE Cornwall, United Kingdom;; ^b^Department of Biology, Emory University, Atlanta, GA 30307

**Keywords:** CRISPR-Cas, bacteria, phage, evolution

## Abstract

Articles on CRISPR commonly open with some variant of the phrase “these short palindromic repeats and their associated endonucleases (Cas) are an adaptive immune system that exists to protect bacteria and archaea from viruses and infections with other mobile genetic elements.” There is an abundance of genomic data consistent with the hypothesis that CRISPR plays this role in natural populations of bacteria and archaea, and experimental demonstrations with a few species of bacteria and their phage and plasmids show that CRISPR-Cas systems can play this role in vitro. Not at all clear are the ubiquity, magnitude, and nature of the contribution of CRISPR-Cas systems to the ecology and evolution of natural populations of microbes and the strength of selection mediated by different types of phage and plasmids to the evolution and maintenance of CRISPR-Cas systems. In this perspective, with the aid of heuristic mathematical–computer simulation models, we explore the a priori conditions under which exposure to lytic and temperate phage and conjugative plasmids will select for and maintain CRISPR-Cas systems in populations of bacteria and archaea. We review the existing literature addressing these ecological and evolutionary questions and highlight the experimental and other evidence needed to fully understand the conditions responsible for the evolution and maintenance of CRISPR-Cas systems and the contribution of these systems to the ecology and evolution of bacteria, archaea, and the mobile genetic elements that infect them.

In 1987, a study by Ishino and colleagues (1) aimed at analyzing the nucleotide sequence of the *iap* (isozyme-converting alkaline phosphatase) gene in *Escherichia coli* serendipitously led to the first-ever description of a CRISPR array. Specifically, they identified 14 repetitive sequences of 29 base pairs (bp) each at the 3′ end of the *iap* gene that were interspersed by 32- to 33-bp variable sequences (1, 2). Over the next years, identification of CRISPR arrays in other gram-negative bacteria, gram-positives, and in Archaea (3–7) triggered a quest to identify their biological function (reviewed in ref. 8). Meanwhile, as more whole-genome sequences became available and CRISPR detection algorithms were developed, it became clear that these arrays of repeating sequences are common in prokaryotes, with estimated frequencies of ∼30 to 40% in Bacteria and 90% in the Archaea (9–13), with clear variation between phyla (14). An important step in understanding the function of CRISPRs was the identification of so-called *cas* genes (for CRISPR associated) that are often found in the neighborhood of CRISPR arrays (15). Bioinformatics analyses expanded the known repertoire of *cas* genes (11, 16–19), shed light on their evolutionary origins (20, 21), and led to a comprehensive classification of *cas* gene combinations into two classes and an increasing number of types and subtypes of CRISPR-Cas systems that differ in key mechanistic aspects (12).

The original idea that CRISPR-Cas is an adaptive immune system came from observations that sequences in CRISPR arrays on the chromosomes of bacteria match those of phage and other foreign genetic elements (10, 22, 23). *Cas* genes were—based on their domains and predicted catalytic activities—suggested to encode the protein machinery that carries out the various steps of the immune response (11). The first experimental evidence in support of this hypothesis came from a joint effort by industrial and academic partners, who showed that the lactic acid bacterium *Streptococcus thermophilus* acquired postinfection resistance (hereafter immunity) against viral infection by inserting sequences from the viral genome into CRISPR loci on the bacterial genome (24). Viruses in turn were found to overcome CRISPR-based immunity by mutation of the target sequence on their genome (25). Together, these findings fueled models of how bacteria with CRISPR systems and their viruses might coevolve (26–28). In parallel with this experimental work, genomic data suggested that CRISPR loci evolve rapidly in natural populations of acidophilic bacteria (29) and that the DNA sequences between these palindromic repeats, spacers, were homologous to that of phage, plasmids, and transposons (30).

Since these pioneering studies, spacer uptake from phages and other mobile genetic elements (MGEs) in bacteria and archaea from natural and human-associated environments has been inferred from variation in spacer sequences within and between populations of the same species and from their homology to MGE genomes (31–40). Experimental observations of spacer uptake in the laboratory in response to plasmid and phage infection have been made among others in engineered *E. coli* strains (41–43) and *Staphylococcus aureus* (44–47) and in wild-type *Pectobacterium atrosepticum* (48), *Pseudomonas aeruginosa* (49, 50), *Roseburia intestinalis* (51), *Sulfolobus solfataricus* (52), *Streptococcus mutans* (37), and other species (reviewed in ref. 53). Consistent with the hypothesis that CRISPR-Cas protects bacteria from infections, some MGEs encode so-called anti-CRISPR genes that block the activity of the immune system (reviewed in ref. 54).

## How Important Is CRISPR to the Ecology and Evolution of Bacteria and Archaea?

While the preceding evidence and arguments demonstrate that CRISPR-Cas can protect bacteria and archaea from infectious DNAs, it is not at all clear how commonly CRISPR plays this role in natural populations of these microbes. If CRISPR commonly protects bacteria from infectious DNAs, one might expect a strong negative correlation between the presence of a CRISPR system and the signatures of horizontal gene transfer in the same genomes. For restriction-modification systems—the most prevalent innate immune system of bacteria and archaea—such correlations can be readily detected (55). Yet, in the case of CRISPR-Cas the evidence is ambiguous, with some studies suggesting that CRISPR does form a barrier for the movement of mobile genes between microbial species (56, 57), whereas other studies arrive at the opposite conclusion (58–60). Furthermore, most spacers from sequenced isolates have no homology to viral or plasmid sequences in databases (40), and the same holds for spacers extracted from metagenomes (39).

If indeed CRISPR-Cas is commonly important for protecting populations of microbes from phage and preventing the acquisition of MGEs, there should be no trouble detecting (in the laboratory or in nature) CRISPR-Cas–encoding bacteria and archaea that acquire spacers from novel sources of infectious DNAs to become immune to those infections. However, other than the bacterial species listed above, there are very few wild-type bacteria or archaea for which spacer acquisition from phage or plasmids has been demonstrated to occur at observable frequencies. Moreover, even for species that have been reported to acquire spacers, it is not clear whether they do so in response to only few or many phages and plasmids. Because in most bacteria and archaea, spacer acquisition is rare, a range of elegant methods has been developed to detect these events (44, 61–66), and while this has propelled our understanding of the mechanisms of spacer acquisition (53) and CRISPR-mediated immunity (67), it raises questions concerning the ecological importance of CRISPR-Cas immune systems. Perhaps this is because many of the domesticated bacteria and archaea that we use for research simply lost their ability to rapidly acquire spacers. Could it be that the quest to find culturable bacteria, archaea, phage, and plasmids with these properties has not be adequately extensive? Or could it be that the results of these quests are commonly negative and therefore, not reported? Sequence data analysis also provides a mixed picture: CRISPR loci in some species appear to evolve rapidly (e.g., refs. 29, 30, and 32 and reviewed in ref. 68), whereas others are relatively static over long periods (60, 69).

## Open Questions

We hope the preceding has convinced the reader that the following are open questions: (*i*) how commonly CRISPR-Cas systems protect populations of bacteria and archaea from infections with deleterious MGEs and (*ii*) the corollary, that extant CRISPR-Cas systems are maintained by selection mediated by these infectious genetic elements. To address these issues, we explore the answers to the following four questions.

*i*) Under what conditions will immunity be selected for in populations with functional CRISPR-Cas systems (CRISPR^+^ bacteria and archaea) confronted with phage and plasmids?
*ii*) Under what conditions will bacteria and archaea with functional CRISPR-Cas immune systems be able to invade populations that lack CRISPR-Cas systems (CRISPR)?
*iii*) What is the contribution of CRISPR immunity to the population dynamics, ecology, and evolution of prokaryotes and their MGEs?
*iv*) What are the characteristics of MGEs that lead to spacer acquisition by bacteria during an infection?

We explore the first three of these questions in two ways: 1) with heuristic mathematical–computer simulation models of the dynamics of microbes and infectious genetic elements, and 2) with a review of the experimental and other empirical studies that provide some answers to these questions. We separately consider the three main sources of infectious DNAs: lytic phage, temperate phage, and conjugative plasmids. For each of these, we consider the invasion conditions: the conditions under which selection mediated by these infectious MGEs will lead to 1) the ascent of immune cells in populations with functional CRISPR-Cas systems, CRISPR^+^, and 2) the establishment of CRISPR-Cas, CRISPR^+^, in populations that do not have a functional CRISPR-Cas system, CRISPR^−^. The equations for the models used and those employed for the analysis of their properties are presented in *SI Appendix*, as are the caveats and concerns about the limitations of these models and our analyses of their properties.

### Lytic Phage.

#### What the models and theory tell us.

We use a simple model that captures the molecular mechanism of types I and II CRISPR-Cas systems, which are the most abundant systems [30 and 8% of genomes, respectively (70)]. In this model, there is a single population of lytic phage, V; a population of bacteria that lacks a functional CRISPR-Cas system, and a population that carries a functional CRISPR-Cas system (CRISPR^−^ and CRISPR^+^, respectively). The CRISPR^−^ bacteria are of two possible states, phage sensitive S and phage resistant SR, due to phage receptor mutations. The CRISPR^+^ bacteria can be of three states: phage sensitive not immune (C), phage resistant (CR) due to phage receptor mutations, and phage immune (CI) due to the acquisition of a phage-targeting spacer in the CRISPR array (*SI Appendix*, Fig. S1). We assume that resistant bacteria, SR and CR, are refractory to the phage; the phage does not adsorb to these cells. By contrast, immune bacteria, CI, can be infected by the phage, but phages do not replicate and are lost. Resistance is acquired by random mutation and immunity through the acquisition of a spacer by infection with the phage. Both acquisition of a resistance mutation and the acquisition of spacers are stochastic processes, which we model with a Monte Carlo protocol.

**Fig. 1.**
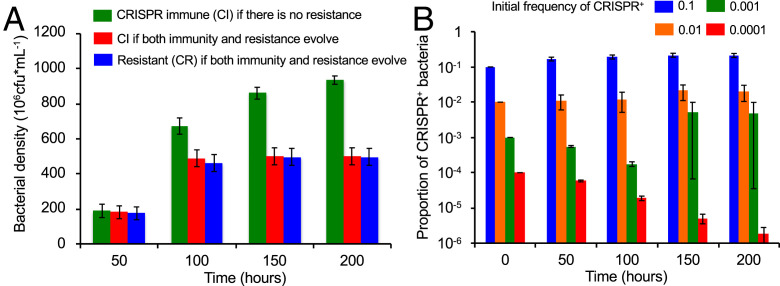
Lytic phage-mediated selection for CRISPR-Cas–mediated immunity and envelope resistance. Invasion conditions. (*A*) Monte Carlo simulations of selection for CRISPR-Cas immunity and resistance in a CRISPR^+^ phage-sensitive population initially at equilibrium with the phage. Mean and SE of the changes in density of CRISPR^+^ immune and resistant bacteria across 100 runs. Green bars are for a population of CRISPR^+^ bacteria that cannot generate resistant mutants. Red and blue bars are for populations that can evolve both CRISPR-Cas immunity (red bars) and envelope resistance (blue bars) with equal probabilities. (*B*) Invasion of CRISPR^+^ into a population of CRISPR^−^-sensitive bacteria at equilibrium with phage, with different initial frequencies of CRISPR^+^ (blue = 0.1, orange = 0.01, green = 0.001, red = 0.0001). Resistant and immune bacteria can be generated in the invading CRISPR^+^ population, and resistance can be generated in the initially dominant CRISPR^−^ population. Mean and SE of the frequency of CRISPR^+^ bacteria over time across 200 runs. Parameters: v_C_ = v_CI_ = v_CR_ = 0.7, δ = 10^−7^, β = 50, e = 5 × 10^−7^, k = 1, RR = 500, w = 0.1, r(0) = 500, µ_SR_ = 10^−8^, µ_RS_ = 10^−8^, *x* = 1.667 × 10^−8^, and the total volume of the vessel is Vol = 100 mL The initial densities of bacteria and phage in these simulations are at the equilibrium for a phage-limited population: C* = 2 × 10^4^ and V* = 6 × 10^6^, respectively.

First, we consider the conditions under which resistance, CR, and CRISPR-Cas–mediated immunity, CI, will invade a CRISPR^+^ phage-sensitive population, C, at equilibrium with the phage. If the CRISPR^+^ population, C, is unable to generate resistant mutants, CR, but can acquire spacers and the likelihood of acquiring a spacer upon infection with the phage is on the order of 10^−8^ or greater, there are broad conditions under which CRISPR-Cas will be selected for and immune cells, CI, will become established in a CRISPR^+^ population. This can be seen from the change in the mean densities of immune cells in Fig. 1*A*. If both immunity and resistance can be generated, both will become established (Fig. 1*A*). Examples of the associated population dynamics are in *SI Appendix*, Fig. S2 *A*–*C*. In the simulations presented in Fig. 1*A*, upon first encounter with the phage, immunity and resistance are equally likely to be generated, and therefore, resistant and immune cells are equally likely to ascend to dominate the CRISPR^+^ population (we assume no costs of resistance). If immunity is 10-fold more likely to be generated than resistance, immune cells are more likely to dominate the population (*SI Appendix*, Fig. S2*D*).

The conditions for bacteria with CRISPR-Cas (CRISPR^+^) to become established in a CRISPR^−^ population of sensitive bacteria at equilibrium with a lytic phage are more restrictive than those for CRISPR-Cas–mediated immunity to become established in a population of CRISPR^+^ bacteria. When the frequency of the invading population of CRISPR^+^ cells is low, 10^−4^ or less, the CRISPR^+^ population does not become established (Fig. 1*B*). Moreover, in only 2 of the 200 runs did the CRISPR^+^ population invade when the initial frequency of CRISPR^+^ was 10^−3^. The reason for the difficulty of CRISPR^+^ bacteria to become established in CRISPR^−^ populations when bacteria with this immune system are initially rare is that, when confronted with phage, resistant mutants are likely to be generated in the dominant population of CRISPR^−^-sensitive cells (*SI Appendix*, Fig. S3 *A*–*C*). The conditions for the invasion of CRISPR^+^ in a CRISPR^−^ population are greater if the rate of spacer acquisition is higher or if the CRISPR^−^ population is unable to generate resistant mutants (*SI Appendix*, Fig. S3 *D* and *E*)

The invasion theory considered above addresses only one element of the role of phage in the evolution of CRISPR-Cas. The other element is the length of time selection mediated by lytic phage will favor CRISPR-Cas immune systems. This will depend on 1) the capacity of the phage to generate protospacer mutants, 2) the capacity of the bacteria to acquire novel spacers to counter protospacer mutations in the phage, 3) the rates at which these novel spacers are acquired, 4) the rate of mutation to resistance, and 5) the fitness costs associated with the carriage of CRISPR-Cas, surface resistance, and protospacer mutations in the phage. Some theoretical studies have partly addressed this issue (27, 28, 71), but the models employed do not consider how all five of the above-listed factors contribute to the length of time selection for CRISPR-Cas will be maintained.

#### What the experiments and genomic data say.

While we are unaware of experimental studies that examine the invasion of CRISPR^+^ into established CRISPR^−^ populations of bacteria or archaea, there have been several experimental studies of the population and evolutionary dynamics of lytic phage and bacteria with CRISPR-Cas systems. As anticipated by the models, CRISPR-Cas immunity readily evolves in *S. thermophilus* strains exposed to virulent phage (24, 72). In this system, bacteria with envelope resistance are normally not detected, and an extended spacer–protospacer arms race can ensue when these bacteria and phage coexist in serial transfer culture (24, 73–75). While the phage will eventually be lost, the duration of the arms race and the diversity of spacers and phage protospacer mutants that evolve during this process can be substantial. In these experimental populations, the densities of bacteria remain at levels similar to those of phage-free populations. Stated another way, the bacterial populations are limited by resources, rather than the phage.

In these experiments, it is clear that the coevolutionary dynamics observed for *S. thermophilus* and its phage can be attributed to CRISPR-Cas–mediated immunity to the phage. Resistant bacteria only evolve if the CRISPR-Cas system is inactivated by either antisense RNA expression (76) or an anti-CRISPR (*acr*) gene encoded by the phage (77), with resistance due to mutations in either the receptor or intracellular host genes required for completing the phage life cycle. This system, therefore, corresponds well with the theoretical scenario in Fig. 1*A* (green bars), which therefore may explain why the evolution of CRISPR immunity is so commonly observed in this model organism.

Also consistent with the theoretical predictions are the results of experiments with bacteria that can evolve both CRISPR immunity by the acquisition of spacers and resistance by mutation of the phage receptors. For example, *P. aeruginosa* strain PA14 either evolves resistance against phage DMS3*vir* (a temperate phage locked in the lytic cycle) by mutation of the type IV pilus or immunity by the acquisition of spacers into its CRISPR arrays (49, 50). Experimental manipulation of the bacterial mutation rate shows that which of these two defense mechanisms prevails during short-term infection studies strongly depends on the rate at which receptor mutants are generated in the population (78). Similarly, the rates of spacer acquisition matter: bacteria with “primed” CRISPR-Cas systems acquire spacers at a higher rate compared with bacteria with naïve CRISPR-Cas systems; this translates in a strong increase in the proportion of bacteria that evolved CRISPR-Cas immunity following phage exposure (41, 42, 48, 61, 79–82). This phenomenon was observed first for many type I where it relies on an imperfect match between a preexisting spacer and the infectious genome (ref. 53 has mechanistic details), and more recently, a similar mechanism was also observed for type II CRISPR-Cas systems (47, 79). When bacteria are exposed to defective phage or when bacteria carry both a restriction modification and a CRISPR-Cas system, the rates of spacer acquisition are also elevated, and again, this leads to higher levels of evolved CRISPR immunity in short-term experiments (83, 84). The typically low frequencies of CRISPR immunity that many bacteria evolve in the laboratory may therefore be at least in part explained by the high mutation rates and large population sizes relative to the rates of spacer acquisition in many model systems, although the fitness costs and benefits of CRISPR-based immunity and surface-based resistance will also be important (50, 85), especially in the long term, which has been reviewed elsewhere (86).

### Temperate Phage.

#### What the models and theory tell us.

In our model (depicted in *SI Appendix*, Fig. S4), there is a single population of free temperate phage, P, and five populations of bacteria. Bacteria that lack a functional CRISPR-Cas system, CRISPR^−^, can exist in two states: susceptible nonlysogens, S, and lysogens, L. Bacteria that carry a functional CRISPR-Cas system, CRISPR^+^, can exist in three states: sensitive nonlysogens, C; lysogens, CL; or CRISPR immune, CI. Infections occur at random at a rate proportional to the product of the densities of free temperate phage, P, and the bacteria. Lysogens and CRISPR immune bacteria can be infected by free phage, but the phage does not replicate and is removed from the free phage population. Upon infection of CRISPR^−^-sensitive cells, S, a fraction λ (0 ≤ λ ≤1) produces lysogens, L. The remaining (1 − λ) infections result in lysis of the host to generate free phage (i.e., the lytic cycle) (87). Infections of CRISPR^+^-sensitive cells, C, have three possible outcomes: a fraction λ (0 ≤ λ ≤ 1 − *x*) produces lysogens, CL with the remaining (1 − *x* − λ) producing lytic infections, and killing the infected host. By the acquisition of a spacer from the phage, a fraction *x* (0 ≤ *x* ≤ 1) of the infections produces CRISPR-Cas immune cells, CI. In addition, at a rate *y* (0 ≤ *y* ≤1), CRISPR-Cas immune cells are generated by lysogens, through the acquisition of a spacer from the prophage. For this analysis, we are not considering lytic mutants of the temperate phage or envelope-resistant mutants.

**Fig. 2.**
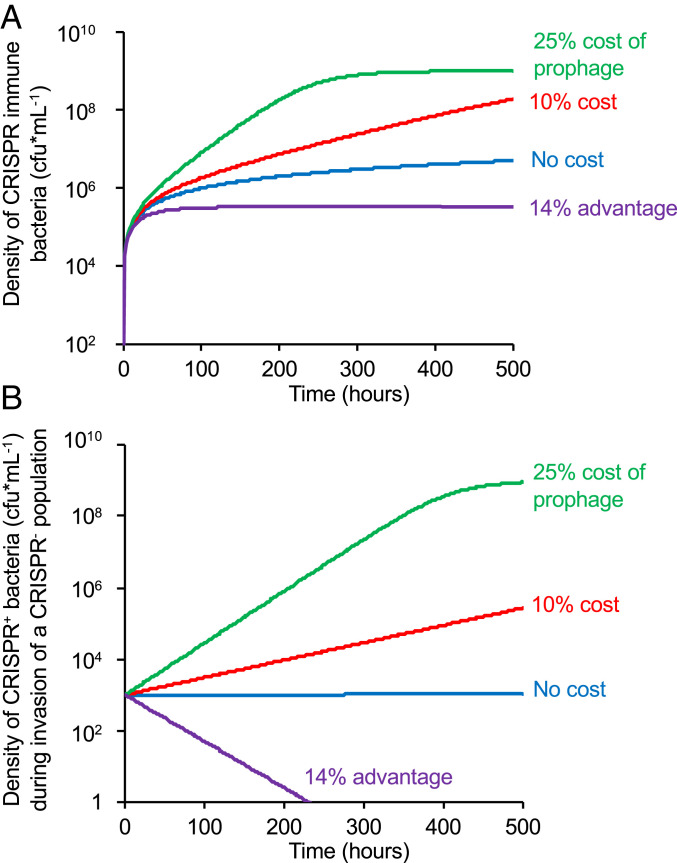
Selection mediated by temperate phage. (*A*) The establishment of CRISPR immunity in a population of CRISPR^+^ bacteria at equilibrium with a temperate phage. Changes in the density of CRISPR-Cas immune bacteria are depicted [colony forming units per milliliter (cfu*ml^−1^)]. (*B*) The invasion of CRISPR^+^ bacteria into a population of CRISPR^−^ bacteria at equilibrium with a temperate phage. Changes in the densities of CRISPR^+^ bacteria are depicted. Carrying the prophage is associated with a 14% advantage, a 10% cost, a 25% cost, or no cost, as indicated. The parameter values are *SI Appendix*, Fig. S5 *C* and *F*.

In Fig. 2 and *SI Appendix*, Fig. S5, we consider the conditions in which selection mediated by temperate phage will lead to the establishment of CRISPR-Cas immunity in a CRISPR^+^ population and the establishment of bacteria with CRISPR-Cas (CRISPR^+^) in a CRISPR^−^ population. During the early stages of infection, temperate phages mostly transmit horizontally through the lytic cycle, and selection for CRISPR immunity will therefore initially be similar to that observed for the lytic phage (Fig. 1 and *SI Appendix*, Fig. S5*A*). Following this, a large subpopulation of lysogens will form that coexists with bacteria that acquired CRISPR immunity in response to horizontally transmitting phage (88). To explore the long-term selection pressures for CRISPR immunity and invasion of CRISPR^+^ into CRISPR^−^ populations, we start our simulations when the bacterial populations are at equilibrium with the temperate phage and therefore, dominated by lysogens (*SI Appendix*, Fig. S5*B*). Under those conditions, for selection mediated by temperate phage to lead to the establishment of CRISPR-Cas immune cells in populations with functional CRISPR-Cas systems, either lysogens have to generate immune cells or the prophage has to reduce the fitness of lysogens relative to nonlysogens (Fig. 2*A*). The rate of ascent of the immune cells is inversely proportional to the fitness of the lysogens. For selection mediated by temperate phage to lead to the invasion of CRISPR^+^ bacteria in a CRISPR^−^ population, the fitness of lysogens needs to be less than or equal to that of nonlysogens. If the carriage of the prophage augments the fitness of the bacteria, as may be the case (89, 90), CRISPR immune bacteria will be selected against, and the CRISPR^+^ population will not be able to invade a population without this immune system (Fig. 2*B*) (88).

#### What the experiments and data say.

There is evidence that CRISPR-Cas systems target temperate phage in nature. For example, spacers encoded by *P. aeruginosa* isolates with type I CRISPR-Cas systems from cystic fibrosis lungs were found to frequently target related groups of temperate phages (including DMS3). Surprisingly, however, in these patients, no spacer acquisition was observed over time (60). By contrast, in an experimental study where a wound model was infected with a mix of six *P. aeruginosa* strains, CRISPR immunity was found to evolve in *P. aeruginosa* strain PA14 against a prophage carried by one of the strains, known as strain B23-2 (91). Another recent study with *P. aeruginosa* PA14 and its phage DMS3 showed that carrying a primed CRISPR-Cas immune system is, in fact, maladaptive during temperate phage infection, due to immunopathology in CRISPR^+^ lysogens since the partial matching spacer triggers cleavage of the prophage. The associated fitness costs caused a rapid invasion of spontaneous mutants that had lost their CRISPR-Cas immune system (88). Acquisition of perfectly matching spacers in lysogens amplified these fitness costs since this programs the immune system to attack the prophage inside the bacterial genome even more strongly. Such self-targeting by CRISPR-Cas is well known to be highly toxic (92–99), even for type III CRISPR-Cas systems that target only transcriptionally active DNA (100, 101). Finally, a recent and exciting study showed that *R. intestinalis* in the mouse gut can evolve high levels of CRISPR-based immunity when one of its active prophages evolves to become hypervirulent (i.e., virulent phage mutants that can infect the lysogen) (51). This, however, brings us to what is anticipated for lytic phage. We are unaware of empirical studies that have explored the contribution of temperate phage to the establishment of CRISPR-Cas in CRISPR^−^ populations.

### Conjugative Plasmids.

#### What the models and theory tell us.

In our model of the population dynamics of CRISPR-Cas immunity and conjugative plasmids, there are five populations of bacteria (*SI Appendix*, Fig. S6). Two lack a functional CRISPR-Cas system, are CRISPR^−^, and can exist in two states: one carrying the plasmid and one not (DP and S, respectively). Three populations carry a functional CRISPR-Cas system, CRISPR^+^, and exist in three states: plasmid free, C; plasmid bearing, CP; and one that is plasmid free but immune to the acquisition of the plasmid, CI. Plasmid transfer is by conjugation, which occurs via random contact between plasmid-bearing and plasmid-free cells (102). A “mating” between DP or CP and S produces SP. A fraction *x* (0 ≤ *x* ≤ 1) of mating between DP or CP with C produces CP, and the remaining fraction of the mating (1 − *x*) produces cells that remain plasmid free but have acquired CRISPR immunity, CI. These CRISPR-Cas immune cells do not take up the plasmid.

The population dynamics of temperate phage and conjugative plasmids are different (87, 102) (compare the temperate phage dynamics in *SI Appendix*, Fig. S5 *A* and *D* with the plasmid dynamics in *SI Appendix*, Fig. S7 *A* and *D*). On the other hand, the conditions under which these infectiously transmitted genetic elements will favor the invasion of CRISPR-Cas immune bacteria into CRISPR^+^ populations and CRISPR^+^ bacteria into CRISPR^−^ populations are virtually identical (*SI Appendix*, Fig. S7 *C* and *F*). The reason for this is that simulations are initiated at equilibrium where cells carrying the MGEs are the dominant population. Whether CRISPR-Cas immune cells will invade and become established in populations with lysogens and bacteria carrying conjugative plasmids depends on the fitness cost of these genetic elements: the greater the cost, the broader the conditions for the establishment and maintenance of these elements. As with temperate phage, if the plasmid increases the fitness of the bacteria, CRISPR-Cas immunity would be selected against (103).

#### What the experiments and data say.

Experimental studies demonstrate that bacteria can evolve CRISPR-based immunity against plasmids, and in the case of type I CRISPR-Cas systems, spacer acquisition is accelerated if the CRISPR immune system is primed. Most commonly, this priming is accomplished by engineering the plasmid in a way that it contains a sequence with a partial match to a preexisting spacer on the genome of a CRISPR^+^ host (41, 48, 61, 104). As anticipated by the model, CRISPR immunity will be selected against if the plasmid provides a net benefit to the host: for example, when it confers resistance to an antibiotic that is present in the environment. Rare mutants that lack an intact CRISPR-Cas immune system will quickly replace the dominant CRISPR^+^ population (105). The model predicts that if the carriage of the plasmid engenders a fitness cost, CRISPR-mediated immunity to that plasmid will be favored. To our knowledge, this has not been demonstrated experimentally. Indeed, the evidence we are aware of is inconsistent with this hypothesis. A study with engineered *E. coli* strains showed that even under these conditions, CRISPR immunity can be maladaptive because the time between infection and clearance of the plasmid may allow for the expression of toxin/antitoxin cassettes. This, in turn, triggers a significant cost of plasmid removal because (short-lived) antitoxin is no longer produced to neutralize the long-lived toxin molecules (106). How common this is, however, is not at all clear. We anticipate that CRISPR immunity will be favored when the plasmid engenders a fitness cost, provided that any costs of plasmid removal do not outweigh the benefits of being plasmid free. We are unaware of any experimental or other empirical studies that have addressed the question of the conditions under which plasmid-mediated selection will favor the invasion of bacteria with CRISPR-Cas systems into CRISPR^−^ populations.

### Other Reasons Why Virulent Phage May Not Select for CRISPR Immunity.

In the heuristic model considered here and the experiments described in the preceding section, the acquisition of spacers confers immunity to the MGE from whence the spacers are derived. This may not always be the case. More and more MGEs are found to encode anti-CRISPR (*acr*) genes that suppress CRISPR-Cas immune systems through a range of different mechanisms and with often high specificity for a single subtype (107–116). The ability of *acr* genes to bypass immunity of bacteria that are already CRISPR immune varies (117, 118), but even the weakest *acr* genes characterized to date effectively block the evolution of CRISPR immunity (119).

Other viruses can bypass CRISPR immunity without a need for *acr* genes. Some “jumbophages,” which are a class of phages with genome sizes that exceed 200 kb, have been reported to form nucleus-like structure during infection (120–122). These structures contain the phage genomes, which shield them from DNA-targeting CRISPR-Cas systems but not from systems that have RNA-targeting activity, such as types III-A and VI-A CRISPR-Cas (123, 124). This variation in the level of protection explains why in nature spacer acquisition from nucleus-forming jumbophages is detected more frequently for bacteria that carry type III systems compared with those that carry type I-E or I-F systems (123). A lack of protection by CRISPR immunity is not limited to jumbophages: *E. coli* strains carrying type I-E CRISPR-Cas that were engineered to carry a single targeting spacer against different phages revealed a lack of protection against phages R1-37 (a giant phage) and T4 (125). The ability of phage T4 to bypass type I-E CRISPR immunity is at least in part attributable to their genome containing glucosyl-5-hydroxymethylcytosine instead of cytosine (126), and this cytosine modification also confers infectivity to the phage when bacteria have type II-A CRISPR-based immunity (126, 127) but not when they have type V-A CRISPR-based immunity (126). Type I-E CRISPR-Cas offers protection against phage T7 but only under low phage densities; at high multiplicity of infection, the cultures were lysed as efficiently as uninduced controls. Efficient protection against T5 was only observed if the CRISPR spacer targeted a pre-early gene (125). Furthermore, a recent study demonstrated that a type I-F CRISPR-Cas system of *P. atrosepticum* reduced the efficiency of plaquing of two virulent phages ϕTE and ϕM1 when the immune system was engineered to carry spacers targeting these phages. Interestingly, CRISPR-Cas was unable to rescue the host from cell death, hence resulting in an abortive infection-like phenotype that blocks phage propagation (128). It remains to be determined if and when CRISPR immunity would evolve in bacterial populations exposed to these phages.

These observations are in stark contrast with the high levels of protection against virulent mutants of temperate phage. Examples include high levels of CRISPR-based immunity observed in *P. aeruginosa* strain PA14 against its phage DMS3vir, which we discussed above. Mild or strong overexpression of the type I-E CRISPR-Cas system of *E. coli* targeting the nonlysogenic mutant λvir provides full immunity, with efficiency of plaquing around 10^−6^ (125, 129). Similarly, the type II-A CRISPR-Cas immune system of *Streptococcus pyogenes* SF370 provides high levels of immunity when expressed in *S. aureus* RN4220 against the staphylococcal phage ϕNM4γ4, a lytic mutant of ϕNM4 (44–46), and the type III-A from *Staphylococcus epidermidis* RP62a provides high levels of immunity when expressed in *S. aureus* RN4220 against phage ϕNM1γ6, a lytic mutant of the temperate phage ϕNM1 (101). Mapping the variability in the levels of protection conferred by CRISPR-Cas immunity using a wider range of CRISPR immune systems and phages will be critical to understand when and where these systems matter.

## Consequences of CRISPR-Cas Immunity to the Population Dynamics, Ecology, and (Co-)Evolution of Bacteria and Lytic Phage

In experiments where bacteria evolve high levels of CRISPR-based immunity, three possible outcomes have been observed: the phage 1) is being eliminated in short order, 2) persists for an extended time in spacer–protospacer arms race but is eventually to be lost, or 3) persists without coevolution. The first outcome is observed when *P. aeruginosa* PA14 is infected with DMS3*vir* (130, 131). This is because these bacteria acquire many different spacers at the population level, which increases the degree of protection since it reduces the evolution and spread of phage that overcome host immunity (88, 130, 132), which they can do by point mutation (25) or deletion of the target regions on the phage genome (133). A spacer–protospacer arms race is observed when the spacer diversity at the population-level is reduced. In this case, the phage can evolve to overcome host immunity, and hosts, in turn, need to acquire novel spacers to regain immunity. This coevolutionary interaction is observed when *S. thermophilus* is infected by the lytic phage 2972: bacteria and phage coexist and coevolve for an extended time eventually for the phage to be lost (73, 75, 134). Phage extinction is in this system due to the arms race being asymmetrical: acquisition of novel spacers is cost free for the host (135), whereas accumulating point mutations reduces the fitness of the phage (136). Moreover, the host population gradually increases the diversity of spacers, which makes it harder for the phage to keep up with the host (75). Finally, the phage may continue to be maintained without coevolution, when bacteria with CRISPR immunity in the population continuously acquire mutations in their CRISPR-Cas immune systems that cause phenotypic reversion to sensitivity (74) or when there is a continuous influx of sensitive bacteria due to immigration (137). While important progress has been made in understanding the consequences of the evolution of CRISPR immunity, most studies have been carried out in highly simplified environments with a single host species infected with a single phage in well-mixed and nutrient-rich broth. Future studies that examine these interactions under more ecologically relevant conditions are desperately needed to understand how CRISPR-Cas systems shape microbial population and evolutionary dynamics in nature.

## Conclusion and Future Directions

We do not question the validity of the hypothesis that CRISPR-Cas systems can protect bacteria and archaea from infections with deleterious MGEs. However, what remains unclear is the magnitude of the contribution of these systems to the ecology and evolution of populations of bacteria and archaea and their phage and other MGEs. As outlined above, many key questions remain. First, more experimental and observational studies are needed to understand not only how frequently but also when, where, and why CRISPR-Cas systems play a role in defense against MGEs (138). Second, it remains unclear how commonly selection mediated by MGEs is responsible for the existence and maintenance of CRISPR-Cas systems in populations of bacteria and archaea and how this is determined by the type of MGE. Third, while CRISPR-Cas systems clearly spread by horizontal gene transfer, it remains unclear how these genes are able to invade a population from rare, especially if the bacteria or archaea can evolve envelope resistance as well. Finally, our understanding of the ecological and evolutionary consequences of CRISPR-Cas immune responses is limited to in vitro experiments that lack much of the biotic and abiotic complexity of natural environments. Could it be that the biotic and abiotic complexity of the real world, where communities of microbes include multiple species and strains as well as diversity in phage and plasmids, are spatially structured, and exist in fluctuating environments, is critical to the evolution and maintenance of CRISPR (50, 85, 139–141)? Filling these gaps in our current understanding of CRISPR ecology and evolution requires interdisciplinary approaches that combine observational studies and mathematical and computer simulation models, as well as population and evolutionary dynamics experiments. The question is how can we do experiments in a way that they would also provide a test of the generality of the hypotheses that are being examined. For that, we would need a diverse array of culturable bacteria and archaea with functional CRISPR-Cas systems and a diverse set of phage and plasmids, which inevitably require many different research teams to examine these questions. This brings us back to our concern about the dearth of bacteria and archaea phage and plasmid systems amenable for these experimental studies and the “fishing expedition” dilemma that a quest to find new systems engenders. However, it is always more difficult publishing negative evidence, no matter how informative that evidence would be. We argue that there is a pressing need to publish any negative results of spacer acquisition in response to MGEs since knowing which culturable bacteria and archaea with functional CRISPR-Cas do and do not acquire spacers and how this depends on the type of infectious DNA will be critical to fully understand the evolutionary ecology of CRISPR-Cas.

## Supplementary Material

Supplementary File

## Data Availability

All study data are included in the article and *SI Appendix*.
