# Supplementary material for: It is unclear how important CRISPR-Cas systems are for protecting natural populations of bacteria against infections by mobile genetic elements
Source: Proc Natl Acad Sci U S A. 2020 Oct 29;117(45):27777–85. doi: 10.1073/pnas.1915966117 (PMC7668106; doi:10.1073/pnas.1915966117)
Supplement: Supplementary File [file pnas.1915966117.sapp.pdf]

It is unclear how important CRISPR-Cas systems are for protecting natural populations of bacteria against infections by mobile genetic elements

Edze Westra and Bruce Levin

Supplemental Material. A theoretical consideration of the population and evolutionary dynamics of CRISPR-Cas mediated immunity

As considered in the body of this perspective, CRISPR-Cas can provide bacteria protection against infections by mobile genetic elements of three major sources, lytic (virulent) phage, temperate phage, and self-transmissible plasmids. In this supplemental material, we use simple mathematical and computer simulation models to elucidate the *a priori* conditions under which these three types infectious mobile genetic elements will select for (i) CRISPR-Cas mediated immunity in populations with functional CRISPR-Cas systems, $CRISPR^+$, and (ii) the ascent of $CRISPR^+$ bacteria in populations without functional CRISPR-Cas systems, $CRISPR^-$. A particular focus of our analysis of the properties of these models are "invasion conditions"; the conditions under which selection mediated by mobile genetic elements will lead to increases in the frequency of CRISPR-Cas immune populations when they are initially rare. As in the body of this report, we separately consider these invasion conditions for populations with functional CRISPR-Cas systems, $CRISPR^+$ and populations without these adaptive immune systems, $CRISPR^-$.

For all of these models, we assume the populations are maintained in continuous (chemostat) culture. A limiting resource, r µg/ml from a reservoir where it is present at a concentration R µg/ml enters a habitat of unit volume at a rate w per hour, which is the same rate at which excess resources, bacteria, and free phage are removed. The rate of growth of the bacteria of type i is directly proportional to its maximum growth v_i per cell per hour and hyperbolic function of the concentration of the resource (1).

$$Z_i(r) = v_i \bullet \frac{r}{(r+k)}$$

The parameter, *k,* the Monod constant, is the concentration of the limiting resource, where the rate of growth of the population is half its maximum value. As in (2), we assume the limiting resource is consumed at a rate proportional to the growth rate of those bacteria, $Z_i(r)$, and the amount of resource required to produce a new cell is e µg/ml.

The properties of these models were analyzed numerically with Berkeley Madonna. For copies of these programs and instructions for their use, write to blevin@emory.edu. The models used for these numerical analyses are generic and chosen to illustrate the conditions under which selection mediated by phage and plasmids will favor the evolution of CRISPR-Cas immunity. However, the growth rates, phage infection and lysogeny rates, and plasmid transfer rates are in the ranges estimated in the cited experimental studies.

I. The population dynamics of lytic phage and bacteria with and without CRISPR-Cas immunity

The Model: In this model, depicted in Figure S1, there is a single population of lytic phage, V, and two types of bacteria: those that are CRISPR$^+$ (i.e. carry a functional CRISPR-Cas system) and those that are CRISPR$^-$ (i.e. lack a functional CRISPR-Cas system). CRISPR$^-$ bacteria can exist in two states; they can either be sensitive to the phage or they can have surface resistance, respectively S and SR. CRISPR$^+$ populations can exist in three states: one sensitive to the phage, C, one with surface resistance, CR, and one that is CRISPR immune, CI. The resistant cells, SR and CR, are refractory to the phage, while the CRISPR immune population can be infected by the phage, but the infecting phage are lost. The variables V, S, SR, C, CR, and CI are both the designations and densities of these populations, particles, in cells per ml. The variables and parameters of this and the other models, their dimensions, and for the parameters the values used are listed in Table S1.

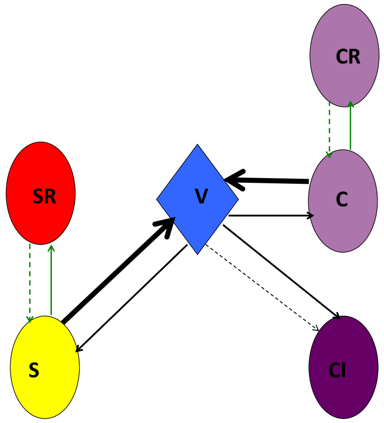

Figure: S1 Lytic phage model: V - free phage, S - CRISPR$^-$ sensitive bacteria, SR - CRISPR$^-$ resistant (refractory) bacteria, C - CRISPR$^+$ phage sensitive bacteria, CR - CRISPR$^+$ resistant (refractory) bacteria, CI - CRISPR$^+$ immune bacteria. The thin black arrows indicate infection and thick black arrow lytic production of phage. The thin green solid and dotted lines designate transitions between states due to mutation or phenotypic processes, respectively, the generation and loss of resistance.

In the absence of phage, bacteria grow at maximum rates, v_S, v_{SR}, v_C, v_R and v_{CI} per cell per hour. The phage adsorb to the bacteria at a rate proportional to the product of their densities and a rate parameter, δ cell x ml/hour (3). If the hosts are sensitive to the phage, S or C, upon infection β phage particles per cell, the burst size, are produced and the infected host cells are removed from the population. Phage infecting $CRISPR^+$ immune cells, CI, are lost and removed from the population. A fraction, x ($0 \leq x \leq 1$) of the adsorptions of phage V to sensitive $CRISPR^+$ cells, C, produce immune cells CI (4). By mutation at a rate μ_{SR} per cell per hour, sensitive cells generate resistant mutants, S→SR, and C→CR. As in (5) we assume that either by mutation or phenotypic processes, at a rate μ_{RS} per cell per hour resistant cells become susceptible, SR→S, and CR→C. With these definitions, the rates of change in the densities of the bacteria and phage and concentration of resources is given by the following set of coupled differential equations.

$$\frac{dr}{dt} = w \bullet (RR - r) - e \bullet \psi(r) \bullet (v_S \bullet S + v_{SR} \bullet SR + v_C \bullet C + v_{CR} \bullet CR + v_{CI} \bullet CI)$$

$$\frac{dS}{dt} = v_S \bullet S \bullet \psi(r) - \delta \bullet V \bullet S + \mu_{RS} \bullet SR - \mu_{SR} \bullet S - w \bullet S$$

$$\frac{dSR}{dt} = v_{SR} \bullet SR \bullet \psi(r) + \mu_{SR} \bullet S - \mu_{RS} \bullet SR - w \bullet SR$$

$$\frac{dC}{dt} = v_C \bullet C \bullet \psi(r) - \delta \bullet V \bullet C - \mu_{SR} \bullet C + \mu_{RS} \bullet CR - w \bullet C$$

$$\frac{dCR}{dt} = v_{CR} \bullet CR \bullet \psi(r) + \mu_{SR} \bullet C - \mu_{RS} \bullet CR - w \bullet CR$$

$$\frac{dCI}{dt} = v_{CI} \bullet CI \bullet \psi(r) + x \bullet \delta \bullet V \bullet C - w \bullet CI$$

$$\frac{dV}{dt} = \delta \bullet V \bullet S \bullet (\beta - 1) + \delta \bullet V \bullet C \bullet (1 - x) \bullet (\beta - 1) - \delta \bullet V \bullet CI - w \bullet V$$

$$\textit{where} \quad \psi(r) = \frac{r}{(r+k)}$$

<u>The evolution of CRISPR immunity and surface resistance in $CRISPR^+$ populations</u>

When sensitive populations of $CRISPR^-$ bacteria, S, are confronted with phage, resistant mutants, SR, will ascend and become the dominant bacterial population. In $CRISPR^+$ populations this dominant population will be either resistant bacteria, CR, or immune cells, CI, which are produced by the acquisition of a spacer from the phage. Whether resistant mutants

or CRISPR-Cas immune cells will dominate depends on the rate of mutation to resistance, S→SR and C→CR, and the rate of spacer acquisition, C→CI.

To illustrate this, we consider a continuous culture community of sensitive bacteria of density N cells per ml at equilibrium with lytic phage in a habitat where resources are sufficient for the rates of growth to be at their maximum. Under these conditions, the densities of the phage and bacteria populations would be,

$$V^* = \frac{(v-w)}{\delta} \text{ and } N^* = \frac{w}{\delta \bullet \beta}$$

Where V^* is the equilibrium density of the phage and N^* the equilibrium density of the bacteria. For example, if the maximum growth rate of the bacteria, v=0.7 per hour, the adsorption rate constant, $\delta = 10^{-7}$, the burst size β=50, and the flow rate w=0.1 per hour, the equilibrium densities of bacteria and phage would be, respectively $N^* = 2.0 \times 10^4$ and $V^* = 6.0 \times 10^6$.

For a population of $CRISPR^+$ sensitive bacteria at equilibrium with phage, C* and V*, whether resistant, CR, or immune CI cells will appear first will depend on the rate of mutation to resistance, μ_{SR}, the likelihood of the bacteria picking up a spacer, the parameters δ and x and the total number of bacteria, C*•Vol. If $\mu_{SR} \bullet C^* \bullet Vol > x \bullet \delta \bullet V^* \bullet C^* \bullet Vol$, resistance will more likely appear before CRISPR immunity. In general, for CRISPR-Cas immune cells to be generated before resistant ones,

$$x \bullet \delta \bullet V^* \bullet C^* > \mu_{SR} \bullet C^* \text{ or } x > \frac{\mu_{SR}}{\delta \bullet V^*} \quad .$$

For example, if the mutation rate to resistance is $\mu_{SR} = 10^{-8}$, with $\delta = 10^{-7}$ and $V^* = 6 \times 10^6$, for CI to appear before CR, the probability of acquiring a spacer upon infection, x has to exceed 1.67×10^{-8}. If $x = 1.67 \times 10^{-7}$, the probability of acquiring a spacer is 10X as great as that of generating a resistant mutant.

<u>(i) Establishment of resistance and immunity in a CRISPR+ population</u>: In Figure S2, we consider the establishment of immune and resistant bacteria, respectively CI and CR, in a phage sensitive $CRISPR^+$ population, C, initially at equilibrium with the phage. For these simulations, we use a semi-stochastic version of this model where the generation of mutants

or the acquisition of spacers are determined by a Monte Carlo process, with the rest of the transitions between states being deterministic. Consequently, although all of the runs were started with the same conditions, the dynamics differed because mutation and the acquisition of spacers are stochastic processes. In Figure S2A, B, and C we present runs with different outcomes: A where the immune cells become the dominant population, B where the resistant cells become the dominant population, and C where the resistant cells become the dominant population but a high density of immune cells are maintained.

For a more comprehensive perspective of when these different outcomes are anticipated and the likelihood of their occurrence, we use a Monte Carlo simulation to determine the average density of immune and resistant cells at different times for 100 runs with these parameters (Figures 1A and S2D). In the absence of mutation to resistance, $\mu_{SR}=\mu_{RS}=0$, within short order immune cells, CI, evolve and dominate the CRISPR$^+$ population. When resistance and immune cells are equally likely to be generated, both are equally likely evolve to ascend to dominate the bacterial population. If, as in Figure S2D, CRISPR-Cas immunity is more likely to be generated than envelope resistance, immune cells evolve to dominate.

(ii) Establishment of CRISPR$^+$ bacteria in CRISPR$^-$ populations: In Figure S3A-C, we consider the dynamics of the changes in the densities of the different populations of bacteria, S, SR, C, CI and CR, the phage V, and the concentration of the resource, r, for situations where resistance can be generated $\mu_{SR}=\mu_{RS}=10^{-8}$. In these simulations, the sensitive populations are initially at equilibrium with the phage, V= $6x10^6$, and the density of CRISPR$^+$ is around 10% of the total population, respectively C=$2x10^3$ and S=$1.8x10^4$ cells ml^{-1}. CRISPR$^-$, SR, or CRISPR$^+$, CR, cells can emerge and ascend to dominate the population (Figure S3A and B, respectively). CRISPR$^+$ immune cells, CI, can also ascend to dominate the bacterial population (Figure S3C). In this latter simulation the probability of the acquisition of spacer upon infection was 10 times as great as that in Figures S3A and B, x=1.667x 10^{-7} rather than x=1.667x 10^{-8}.

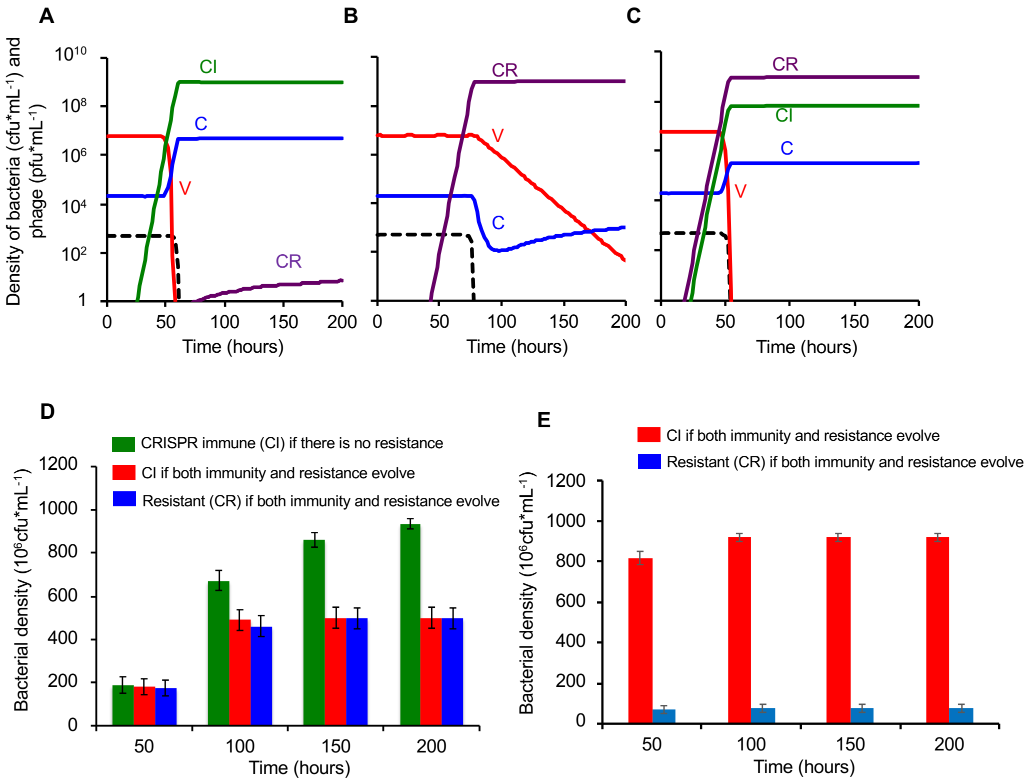

Figure S2: Establishment of immune, CI, and resistant, CR, cells in a sensitive CRISPR$^+$ population, C. Standard parameters v_C=v_{CI}=v_{CR} = 0.7, δ=10^{-7}, β=50, e=$5x10^{-7}$, k=1, RR=500, w=0.1, r(0) =500, μ_{SR}=10^{-8} μ_{RS}=10^{-8}, and the total volume of the vessel is Vol=100 ml. The initial densities of bacteria and phage in these simulations are at the equilibrium for a phage-limited population, respectively, C^*=$2x10^4$, V^*=$6x10^6$, and x=$1.667x10^{-8}$ unless indicated otherwise. Changes in the densities of bacteria and phage, when A) immunity, CI, arises and ascends to dominance before resistance, CR; B) resistance, CR, emerges and ascends to dominance before immune cells are generated or C) resistance emerges and ascends shortly before immune cells emerge. D) The outcome of 100 independent simulations, mean and standard error of the density of CI and CR at different times, where the probability of spacer acquisition x=$1.667x10^{-8}$ (this figure is also shown in the main text, as Figure 1A) or E) x=$1.667x10^{-7}$. Black dotted lines are resource concentration.

To determine the relationship between the initial frequencies of CRISPR$^+$ and CRISPR$^-$ on the conditions for CRISPR$^+$ cells to become established in CRISPR$^-$ population, we use Monte Carlo simulations, 200 runs with each set of parameters. We follow the changes in the density of CRISPR$^+$ cells at different at times for different initial frequencies of CRISPR$^+$ cells. As our criterion for the establishment of CRISPR$^+$ in a CRISPR$^-$ population, we consider the change in the density of CRISPR$^+$ between time 0 and 200 hours. By this criterion CRISPR$^+$ can become established in populations of CRISPR$^-$ when the initial frequency is a low as 10^{-3},

whether immunity is generated at a low or high rate relative to mutation (Figures S3D and S3E). In considering this, it is worth noting that in the runs with the initial frequency of CRISPR$^+$ of 10^{-3}, CRISPR$^+$ dominated at 200 hours in only 2 out of the 200 runs, with both the lower and higher rates of CRISPR-Cas mediated immunity.

Overall, we interpret these simulation results to suggest that if the frequency of CRISPR$^+$ cells that are initially neither resistant or immune to the phage is less than 10^{-3}, the likelihood of CRISPR-Cas immunity successfully becoming established in a CRISPR$^-$ population at equilibrium with the phage is negligible. This is particularly important when considering the establishment of CRISPR$^+$ cells in CRISPR$^-$ populations by horizontal gene transfer when the initial frequency of CRISPR$^+$ cells is going to be low.

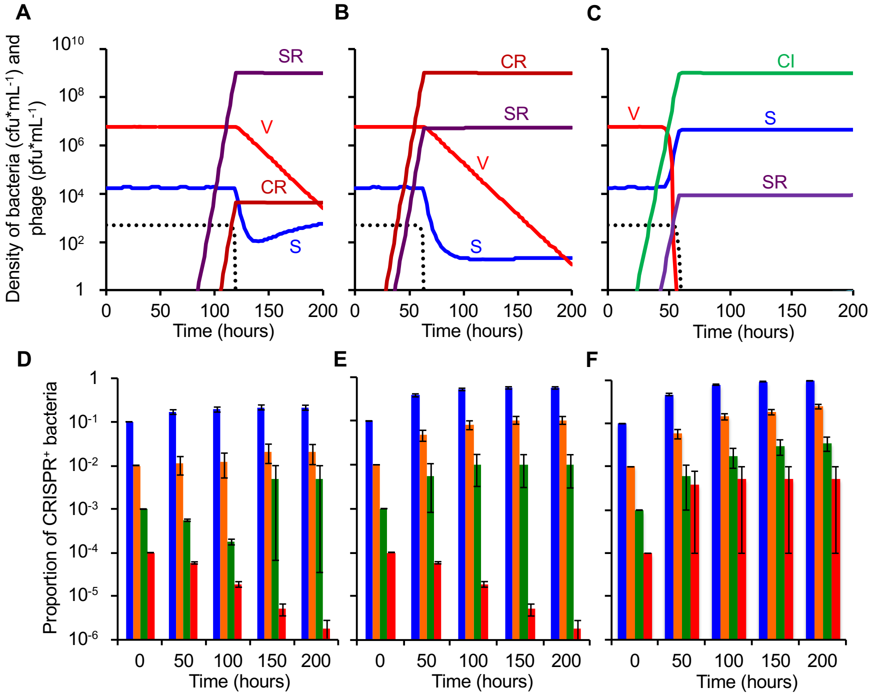

Figure S3. Establishment of CRISPR$^+$ in a CRISPR$^-$ phage sensitive population at equilibrium with lytic phage. Standard parameters, $v_S=v_R=v_C=v_{CI}=v_{CR}= 0.7$, $\delta=10^{-7}$, $\beta=50$, $e=5x10^{-7}$, k=1, RR=500, w=0.1, $\mu_{SR}=10^{-8}$, $\mu_{RS}=10^{-8}$ (save for F) and the total volume of the vessel is Vol=100 ml. In this simulation the equilibrium population of sensitive bacteria includes both CRISPR$^-$ and CRISPR$^+$. A and B) changes in the densities of bacteria and phage in populations initiated with 10% CRISPR$^+$, C, and 90% CRISPR$^-$, S, and $x=1.667x10^{-8}$. A) CRISPR$^-$ resistant cells, SR, evolved to dominate. B) CRISPR$^+$ resistant cells, CR, evolved to dominate. C) $x=1.667x10^{-7}$, and CRISPR$^-$ immune cells, CI,

evolved to dominate. D) Monte Carlo simulations, mean and standard errors in the frequency of CRISPR$^+$ cells in 200 simulated populations initiated with different frequencies of CRISPR$^+$, C, and CRISPR$^-$, S, at equilibrium with the phage with x= 1.667×10^{-8} (this figure is also shown in the main text, as Figure 1B). Blue=0.1, orange=0.01, green=0.001, red=0.0001 initial frequency of CRISPR$^+$. E) Monte Carlo simulations, mean and standard errors in the frequency of CRISPR$^+$ cells. 200 simulated populations initiated with different frequencies of CRISPR$^+$, C, and CRISPR$^-$, S, at equilibrium with the phage with x=1.667×10^{-7}. Color coding as in D. F) Monte Carlo simulations, mean and standard error in the frequency of CRISPR$^+$ cells. 200 simulated populations initiated with different frequencies of CRISPR$^+$, C, and CRISPR$^-$, S, at equilibrium with the phage with x=1.667×10^{-7}, and no resistance possible $\mu_{SR}=\mu_{RS}=0$. Color coding as in D. Black dotted lines are resource concentration.

(iii) Caveats: In choosing the parameters for these simulations of lytic phage selecting for CRISPR-Cas mediated immunity, we are making two assumptions that can affect the predictions. One, all of the populations are equally fit (i.e. they have the same maximum growth rates). For example, if CRISPR-Cas engenders a cost relative to sensitive cells, the conditions for the establishment of immunity in a CRISPR$^-$ or CRISPR$^+$ population are going to be less than that predicted by this model. If CRISPR-Cas is less costly than envelope resistance, $v_{CI} > v_{CR}$, immunity is more likely to evolve than resistance. Two, the populations of sensitive cells being invaded, C or S, are at equilibrium with the phage. If indeed these populations were confronted by phage, they may already be dominated by resistant cells.

II. Population dynamics of temperate phage and bacteria with and without a CRISPR-Cas system.

The Model: In this model, diagrammed in Figure S4, there is a single population of temperate phage, P, and two types of bacteria: those that are CRISPR$^+$ (i.e., carry a functional CRISPR-Cas system) and those that are CRISPR$^-$ (i.e. lack a functional CRISPR-Cas system). The CRISPR$^-$ bacteria can exist in two states; they can either be sensitive non-lysogens, or they can be lysogens (carry the prophage), respectively S and L. The CRISPR$^+$ populations can be present in three states: sensitive non-lysogens, C, lysogens, CL, and CRISPR immune, CI. The phage can exist in three states, as free phage, P, or as prophage in CRISPR$^-$ lysogens, L, or as CRISPR$^+$ lysogens, CL. The bacteria grow at maximum rates, v_S, v_L, v_C, v_{CL}, and v_{CI} per cell per hour. The phage adsorb to the bacteria at a rate proportional to the product of their density,

that of the bacteria, and a rate parameter, δ cell x ml/hour (3). The S and C populations support the lytic replication of the phage. As in (6) with a probability λ ($0 \leq \lambda \leq 1$) upon infection with lytic phage, the S and C populations become lysogens, respectively L and CL. These lysogens are immune to super-infection with the temperate phage, as are the CRISPR$^+$ immune cells, CI; phage that infect these immune cells are removed from the population. CRISPR$^+$ immune cells, CI, are generated in two ways, from existing lysogens, at rate y per cell per hour, and by infection with P, with a probability x per infected cell. In addition to being produced by lytic infections, with a burst size β, free temperate phage are generated by induction of the lysogens, at a rate i per cell per hour. When they lose the prophage, CRISPR$^-$ lysogens revert to sensitivity, S, and when they lose the prophage, CRISPR$^+$ lysogens revert to sensitivity, C. In this model, we assume CRISPR-Cas is not lost.

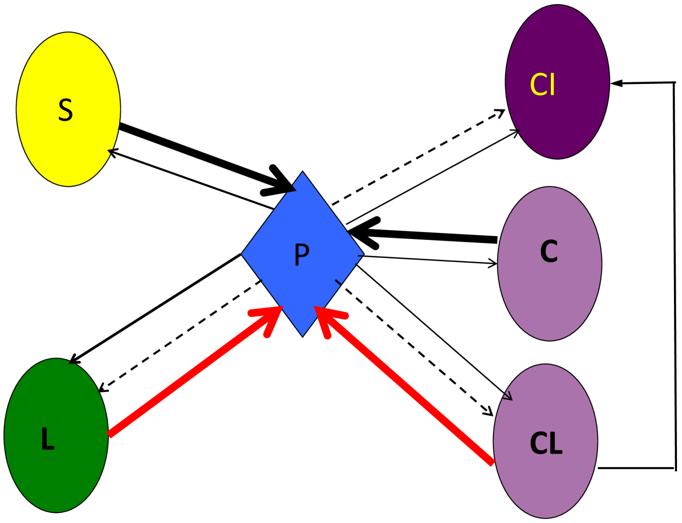

Figure S4. Model of the population dynamics of temperate phage and bacteria with and without CRISPR-Cas systems. P is the density of free phage, S, and L are respectively CRISPR$^-$ sensitive and lysogenic bacteria. C, CL, and CI are, respectively, the designations and densities of CRISPR$^+$ bacteria that are sensitive to the free phage, lysogens, and immune cells. Solid red lines denote the production of free phage by induction of the lysogens. The thick solid black lines denote the production of phage by lytic infection. The thin broken lines denote the loss of the phage due to the adsorption to immune cells, L, CL, and CI. The spacers responsible for the immunity of non-lysogens, C, are picked up by infection with the phage and from CRISPR$^+$ lysogen.

With these definitions and assumptions, the rates of change in the densities of the bacterial populations, free phage, and the concentration of the limiting resource are given by the following set of coupled differential equations.

$$\frac{dr}{dt} = w \cdot (RR - r) - e \cdot \psi(r) \cdot (v_S \cdot S + v_L \cdot L + v_C \cdot C + v_{CL} \cdot CL + v_{CI} \cdot CI)$$
$$\frac{dS}{dt} = v_S \cdot S \cdot \psi(r) - \delta \cdot P \cdot S - w \cdot S$$
$$\frac{dL}{dt} = v_L \cdot L \cdot \psi(r) + \delta \cdot P \cdot S \cdot \lambda - i \cdot L - w \cdot L$$
$$\frac{dC}{dt} = v_C \cdot C \cdot \psi(r) - \delta \cdot P \cdot C - w \cdot C$$
$$\frac{dCL}{dt} = v_{CL} \cdot CL \cdot \psi(r) + \delta \cdot P \cdot C \cdot \lambda - i \cdot CL - y \cdot CL - w \cdot CL$$
$$\frac{dCI}{dt} = v_{CI} \cdot CI \cdot \psi(r) + x \cdot \delta \cdot P \cdot C + y \cdot CL - w \cdot CI$$
$$\frac{dP}{dt} = \delta \cdot P \cdot S \cdot (1-\lambda) \cdot (\beta - 1) + \delta \cdot P \cdot C \cdot (1 - \lambda - x) \cdot (\beta - 1) + i \cdot (L + CL) \cdot \beta - \delta \cdot P \cdot (CI + L + CL) - w \cdot P$$
$$where \;\; \psi(r) = \frac{r}{(r+k)}$$

Temperate phage-mediated selection for CRISPR-Cas: We open our analysis of the properties of this model with an exploration of the conditions under which CRISPR-Cas immune cells, CI, will invade and become established in a CRISPR$^+$ population. In Figure S5A, we consider the dynamics of the invasion of a population of bacteria immune to the temperate phage, CI, in a population initially composed of sensitive non-lysogens and free temperate phage, C and P. Due to lytic infection and the generation of lysogens, the sensitive, C population is lost. The density of the free temperate phage increases rapidly and the C population is converted into lysogens CL. In this simulation, the carriage of the prophage reduces the fitness of the lysogens, CL relative to C and CI. Immune cells, CI, are produced and, because of their fitness advantage over the lysogens, replace the lysogens as the dominant population of bacteria. The density of free phage declines when immune population ascends to dominate. In the absence of CRISPR-Cas mediated immunity, there would be a stable equilibrium with lysogens and temperate

phage, CL and P (6), which can be seen in Figure S5B. In this and the following simulation, Figure S5C, we consider the invasion of CRISPR-Cas immunity, CI, in populations at equilibrium with the temperate phage. In the absence of selection for or against lysogens, the immune cells, CI, increase slowly, due to the conversion of lysogens into immune cells, CL→CI. The rate of increase of the CRISPR-Cas immune cells is proportional to the fitness cost of carrying the prophage (Figure S5C).

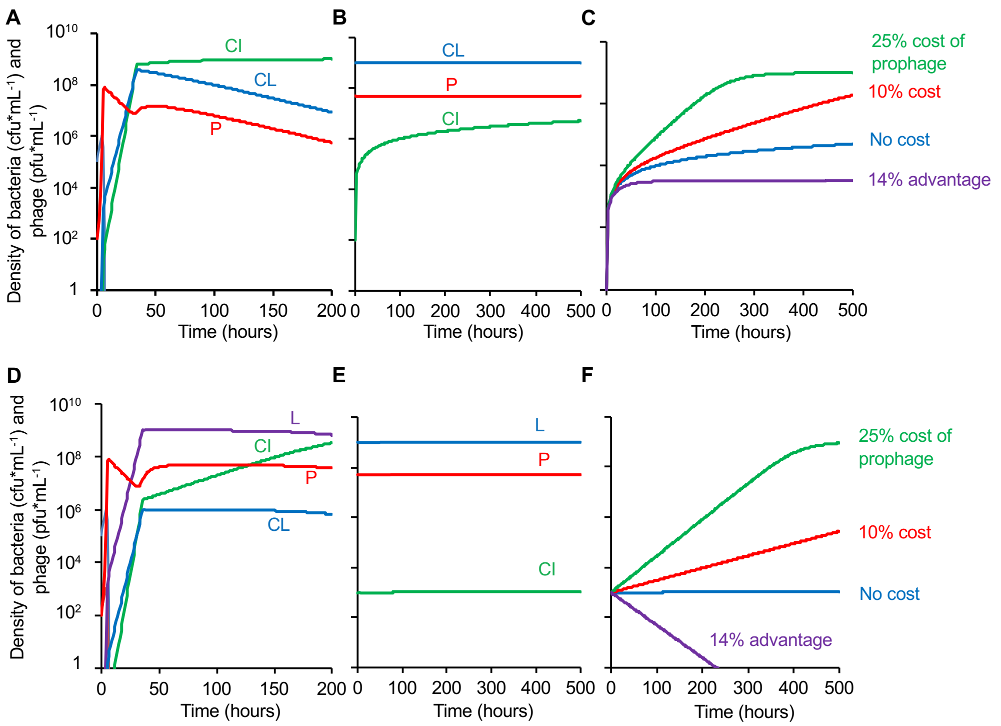

Figure S5 Invasion conditions for CRISPR-Cas mediated immunity in populations of bacteria and temperate phage. Standard parameters: RR=500, w=0.1, k=1.0, e=5x10^{-7}, δ=10^{-7}, β=50, i=10^{-4}, λ=10-3, x=y=10^{-5}. A, B and C) Establishment of immunity in a CRISPR$^+$ population. A) Dynamics of the changes in the densities of free phage, CRISPR$^+$ lysogens, and CRISPR$^+$ immune bacteria, in populations initiated with free phage and sensitive CRISPR$^+$ bacteria, with a 25% fitness cost of lysogens, $v_C=v_{CI}=0.70$, $v_{CL}=0.525$. The light blue line corresponds to the initially sensitive cells, which are quickly lost from the population. B and C) Populations at equilibrium with lysogens and free temperate phage. B) Changes in the densities of bacteria and phage in the absence of selection for or against lysogens, $v_C=v_{CL}=v_{CI}=0.7$. C) Changes in the densities of CRISPR-Cas immune cells with different cost of lysogeny, no cost,

v_C=v_{CI}=0.7, v_{CL}=0.7, a 25% costs v_{CL}=0.525, a 10% cost v_{CL}=0.63, with lysogens 14.3% more fit than non-lysogens v_{CL}=1.0. D, E, and F) Establishment of bacteria with CRISPR-Cas ($CRISPR^+$) in a $CRISPR^-$ population. D) Dynamics of the changes in density of free phage, $CRISPR^-$ and $CRISPR^+$ bacteria in a population initiated with free temperate phage, and $CRISPR^-$ and $CRISPR^+$ sensitive non-lysogens, with a 25% cost due to the carriage of the prophage v_S=v_C=v_{CI}=0.7, v_L=v_{CL}=0.525. The light blue line corresponds to the initially sensitive cells, which are quickly lost from the population. E and F) Population dynamics of $CRISPR^+$ in a population of $CRISPR^-$ bacteria initially at equilibrium with the temperate phage. E) Changes in the densities of bacteria and phage in the absence of selection for or against lysogens v_S=v_L=v_C=v_{CL}=v_{CI}=0.7, F) Changes in the densities of $CRISPR^+$ cells with different costs of lysogeny, no cost v_S=v_C=v_{CI}=0.7, v_L =v_{CL}=0.7, a 25% cost, v_L=v_{CL}=0.525, a 10% cost, v_L=v_{CL}=0.63, a 14.3% advantage v_L=v_{CL}=1.0.

In Figures S5D-F, we consider the invasion of $CRISPR^+$ cells in a $CRISPR^-$ population. In Figure 5D we follow the dynamics of temperate phage, lysogeny and the invasion of $CRISPR^+$ cells (CI and CL) in a population initiated with sensitive non-lysogens, S and temperate phage, P and a 100 $CRISPR^+$ sensitive non-lysogens. The phage density increases initially and, within short order, the $CRISPR^-$ lysogens, L, ascend. While the invading population of $CRISPR^+$ sensitive non-lysogens, C are lost due to the phage, they are converted into lysogens, CL, which are maintained for a while as a minority population. CRISPR-Cas immune cells, CI are generated and, because in this simulation they have a selective advantage over the lysogens, they increase in density, invade, and will eventually become the dominant population of bacteria. In Figure S5E we consider a population of $CRISPR^-$ lysogens at equilibrium with the temperate phage and a minority population of CRISPR immune cells, CI in the absence of selection for or against the lysogens, L and CL. Under these conditions, the density of the invading $CRISPR^+$ population does not increase or decrease. If, the prophage reduces the fitness of lysogens, the $CRISPR^+$ population will invade, Figure S5F with the rate of ascent inversely proportional to the costs. If the prophage augments the fitness of the lysogens, relative to non-lysogens, the $CRISPR^+$ population will be selected against.

III. Population dynamics of conjugative plasmids and bacteria with and without CRISPR-Cas mediated immunity:

The Model: There are two populations of CRISPR$^-$ cells, one that carries the plasmid and one that does not, DP, and S, respectively, and three populations of CRISPR$^+$ cells, plasmid-free, plasmid-bearing, and immune, respectively C, CP, and CI. These populations grow at maximum rates, v_S, v_P, v_C, v_{CP}, v_{CI} per cell per respectively. The plasmids are transferred at rates proportional to the product of the densities of plasmid-bearing and plasmid-free cells and a rate parameter, γ (7). CRISPR$^+$ cells, C, acquire spacers and become immune to infection with the plasmid, CI, at a rate proportional to the product of their densities, the rate constant of plasmid transfer, γ, and the probability of picking up a spacer x ($0 \leq x \leq 1$) upon conjugation. Immune CRISPR$^+$ cells can also be generated from plasmid-bearing CP at a rate y per cell per hour. Plasmids are lost by vegetative segregation at a rate τ per cell per hour, resulting in DP cells reverting back to S and CP cells reverting back to C.

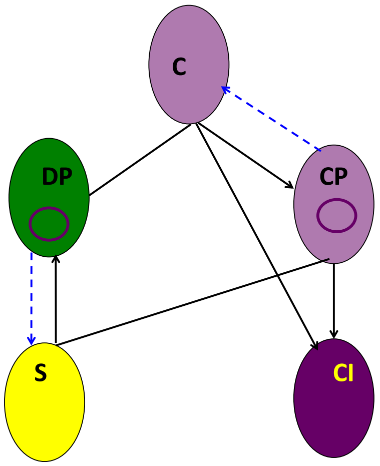

Figure S6 Model of the population dynamics conjugative plasmids and bacteria with and without CRISPR. There are two populations of CRISPR$^-$ cells, plasmid-free, and plasmid bearing, S, and DP. There are three populations of CRISPR$^+$ bacteria, those that are plasmid-free, those that carry the plasmid and those that are immune to the plasmid, respectively, C, CP, and CI. Plasmids are transferred to plasmid-free cells at a rate proportional to the product of their densities and a rate constant, γ. Immune, cells, CI are produced by infection of C by mating with a plasmid bearing cells or from a transition from CP to CI.

With these definitions and assumptions, a chemostat habitat and resource-limited growth, the rates of change in the densities of the different populations and concentration of the resource are given by the following array of coupled differential equations.

$$\frac{dr}{dt}=w\bullet(RR-r)-e\bullet\psi(r)\bullet(v_S\bullet S+v_{DP}\bullet DP+v_C\bullet C+v_{CP}\bullet CP+v_{CI}\bullet CI)$$

$$\frac{dS}{dt}=v_S\bullet\psi(r)\bullet S-\gamma\bullet S\bullet(DP+CP)+\tau\bullet DP-w\bullet S$$

$$\frac{dDP}{dt}=v_{DP}\bullet\psi(r)\bullet DP+\gamma\bullet S\bullet(DP+CP)-\tau\bullet DP-w\bullet DP$$

$$\frac{dC}{dt}=v_C\bullet\psi(r)\bullet C-\gamma\bullet C\bullet(DP+CP)+\tau\bullet CP-w\bullet C$$

$$\frac{dCP}{dt}=v_{CP}\bullet\psi(r)\bullet CP+\gamma\bullet(1-x)\bullet C\bullet(DP+CP)-\tau\bullet CP-y\bullet CP-w\bullet CP$$

$$\frac{dCI}{dt}=v_{CI}\bullet\psi(r)\bullet CI+x\bullet\gamma\bullet C\bullet(DP+CP)+y\bullet CP-w\bullet CI$$

$$where\ \ \psi(r)=\frac{r}{(r+k)}$$

When bacteria carrying conjugative plasmids are introduced into receptive populations of plasmid-free cells in continuous culture, the plasmid can sweep through the population and convert the plasmid-free cells into plasmid bearing (7). These dynamics can be seen in Figure S7A, where we consider the invasion of a CRISPR-Cas immune population, CI, into a population of plasmid-bearing and plasmid-free CRISPR$^+$ cells, C and CP. In these simulations, the plasmid engenders of 25% fitness cost; the immune bacteria, CI ascend and eventually replace the plasmid-bearing cells as the dominant population. With the parameters considered in the simulations, in the absence of immunity, CI, there are stable equilibria between the plasmid-bearing and plasmid-free bacteria (7). In the absence of a cost due to the carriage of the plasmid, the initially rare immune population, CI, increase in density in a CRISPR$^+$ population at equilibrium with the plasmid (Figure S7B). This increase in the density of the invading immune population can be attributed to the production of immune cells by the dominant population of plasmid bearing cells, CP→CI at a rate $y=10^{-5}$ per cell per hour. If $y=0$, the density of this immune population would remain at its initial level. The rate at which the density of immune cells increases is inversely proportional to the fitness cost of carrying the plasmid (Figure S7C). As was the case for where there was no cost associated with the carriage of the plasmid, the increase and leveling off in the density of CI when the plasmid-confers a selective advantage can be attributed to the production of immune cells by the plasmid bearing cells. If not for this, the density of the invading population of immune cells would decline.

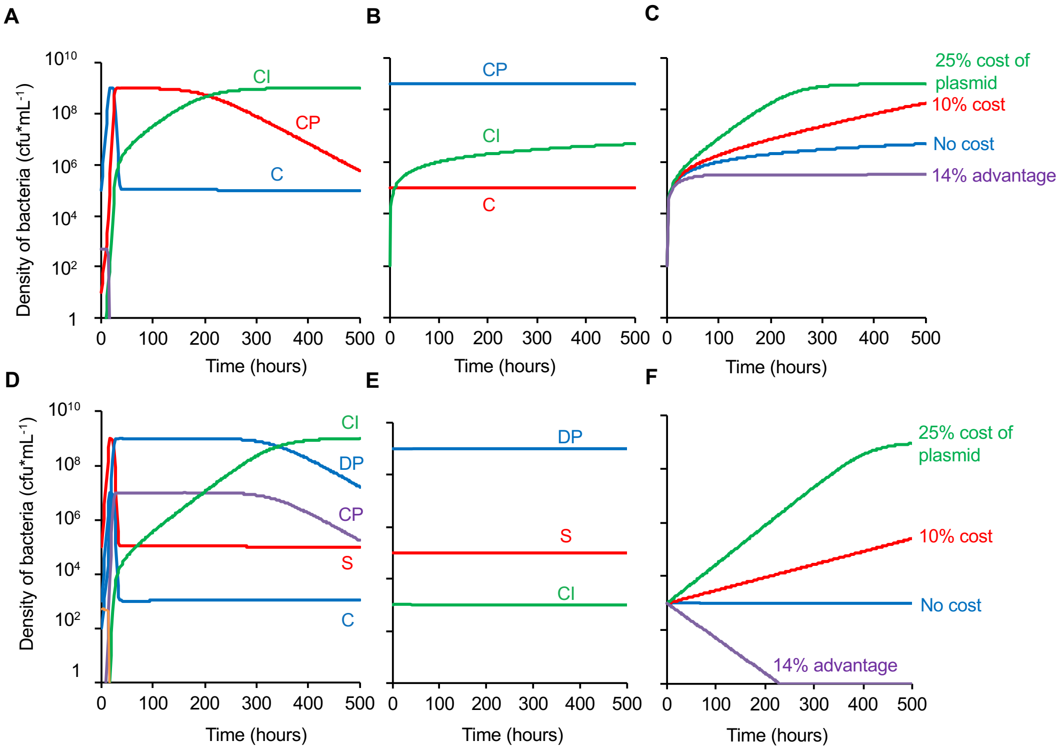

Figure S7. Conditions for the invasion of CRISPR-Cas immunity in populations with conjugative plasmids. Standard parameters, RR=500, w=0.10, e=5×10^{-7}, k=1.0, $\gamma=10^{-9}$, $\tau=10^{-4}$, $x=10^{-4}$, $y=10^{-4}$. Changes in the densities of bacteria. A, B and C) Invasion CRISPR-Cas mediated immunity in a CRISPR^{+} population. A) Dynamics of plasmid transfer and the ascent of a CRISPR-Cas immune, CI, population, when there is a 25% cost of plasmid carriage: $v_C=v_{CI}=0.7$, $v_{CP}=0.525$. B and C) Invasion of CRISPR-Cas immune cells, CI, in population at equilibrium with plasmid bearing and plasmid-free cells, CP. B) Changes in the densities of immune cells, CI, in the absence of selection for or against the carriage of the plasmids: $v_C=v_{CP}=v_{CI}=0.7$. C) Invasion of a CRISPR-Cas immune bacteria, CI, in populations of plasmid-free CRISPR^{+} bacteria with different costs for the carriage of the plasmid: $v_C=v_{CI}=0.7$, $v_{CP}=0.7$ (no cost), $v_{CP}=0.525$ (25% cost), $v_{CP}=0.63$ (10% cost), $v_{CP}=1.0$ (14% advantage). D-F) Invasion of a CRISPR^{-} population by bacteria carrying CRISPR-Cas immune systems (CRISPR^{+}). D) Dynamics of plasmid transfer and the CRISPR^{+} bacteria, CI and CP, in a CRISPR^{-} population with a 25% cost of plasmid carriage: $v_S=v_D=v_C=v_{CI}=0.7$, $v_{DP}=v_{CP}=0.525$. E and F) Invasion of CRISPR^{+} cells in a CRISPR^{-} population at equilibrium with the plasmid, DP and S. E) Changes the densities CRISPR^{-} plasmid-bearing and plasmid free cells, DP and S and a CRISPR-Cas-containing population (CRISPR^{+}) with plasmid immunity, CI, in the absence of selection for or against the carriage of the plasmid. $v_S=v_{DP}=v_C=v_{CP}=v_{CI}=0.7$. F) Changes in the densities of CRISPR^{+} bacteria with different costs of the carriage of the plasmid with different costs for the carriage of the plasmid: $v_C=v_{CI}=0.7$, $v_{CP}=0.7$ (no cost), $v_{CP}=0.525$ (25% cost), $v_{CP}=0.63$ (10% cost), $v_{CP}=1.0$ (14% advantage).

In Figure S7D, we consider the invasion of CRISPR$^+$ cells into a CRISPR$^-$ population with a conjugative plasmid. We follow the changes in the densities of the different populations of plasmid-free and plasmid bearing cells in a community initiated with plasmid-free and plasmid-bearing CRISPR$^-$ bacteria, S and DP, and a minority population of CRISPR-Cas immune cells, CI. In this simulation, the plasmid engenders of 25% fitness cost and the immune bacteria, CI ascend. In Figure S7E and F, we consider the invasions of bacteria with CRISPR-Cas immune system into a population of S and DP at equilibrium in the absence of selection for or against the carriage of the plasmid. Under these conditions, the density of the CRISPR-Cas immune cells, CI remains unchanged. If the plasmid engenders a fitness cost, the immune cells invade at a rate that inversely proportional to the cost of the plasmid (Figure S7F).

The utility and limits of the models

Richard Levins argued that in constructing mathematical models in population biology one has to, sacrifice generality to realism and precisions, sacrifice realism to generality and precision, or sacrifice precision to realism and generality (8). The models developed here are in this last, heuristic, tradition. Their role is to identify the factors that govern the conditions under which CRISPR-Cas immunity will evolve and the relative contributions of these parameters to this evolution. The parameters of these models can be independently estimated in experimental populations of bacteria and archaea with lytic and temperate phage or conjugative plasmids and the hypotheses generated from their analysis tested in experimental populations of bacteria and archaea with phage and plasmids.

Table S1 Parameters and Variables

Common Variables and Parameters	Symbol and dimensions	Value -Range
Resource concentration in the habitat	r μg/ml	0 – 500 μg/ml
Resource concentration in the reservoir	RR μg/ml	500 μg/ml
Volume of the habitat	Vol	100ml
Flow rate into and out of the habitat	w ml per hour	0.10
r when the growth is half its maximum	k μg/ml	1.0 ml
Conversion efficiency (resource/cell)	e - μg/cell	5×10^{-7}
**Lytic Phage Model**		
Density of the phage	V particles per ml	0 - 10^{9}
Density of phage sensitive CRISPR$^-$	S cells per ml	0 - 10^{9}
Density of phage resistant CRISPR$^-$ bacteria	SR cells per ml	0 - 10^{9}
Density of phage sensitive CRISPR$^+$ bacteria	C cells per ml	0 - 10^{9}
Density of phage resistant CRISPR$^+$ bacteria	CR cells per ml	0 - 10^{9}
Density of phage immune CRISPR$^+$ bacteria	CI cells per ml	0 - 10^{9}
Maximum growth rates	v_S, v_R, v_C, v_{CR}, v_I hr^{-1}	0.7
Adsorption rate constant	δ - cells x ml/hour	10^{-7}
Burst size	β particles per cell	50
Probability of the acquisition of a spacer	x per infected cell	1.67×10^{-8}, 1.67×10^{-7}
Mutation rates to resistance and sensitive	μ_{SR}, μ_{RS} per cell per hr	10^{-8}
**Temperate phage Model**		
Density of free temperate phage	P particle per ml	0 - 10^{9}
Density of phage sensitive CRISPR$^-$ bacteria	S cells per ml	0 - 10^{9}
Density of CRISPR$^-$ lysogenic CRISPR$^-$ bacteria	L cells per ml;	0 - 10^{9}
Density of phage sensitive CRISPR$^+$ bacteria	C cells per ml	0 - 10^{9}
Density of CRISPR$^+$ lysogenic bacteria	CL cells per ml	0 - 10^{9}
Density of CRISPR-immune bacteria	CI cells per ml	0 - 10^{9}
Maximum growth rate	v_S, v_L, v_C, v_{CL}, v_{CI} hr^{-1}	0.525 – 1.0
Adsorption rate constant	δ - cells x ml/hour	10^{-8}
Burst size	β particles per cell	50
Probability of the acquisition of a spacer	x per infected cell	10^{-5}
Rate of acquisition of spacers CP→CI	y per cell per hour	10^{-5}
Probability of lysogeny	λ per infection	10^{-3}
Induction rate	i per cell per hour	10^{-4}
**Conjugative plasmid Model**		
Density of CRISPR$^-$ plasmid free bacteria	S	0 - 10^{9}
Density of CRISPR$^-$ plasmid-bearing bacteria	DP	0 - 10^{9}
Density of CRISPR$^+$ plasmid-free bacteria	C	0 - 10^{9}
Density of CRISPR$^+$ plasmid-bearing bacteria	CP	0 - 10^{9}
Density of CRISPR$^+$ plasmid immune bacteria	CI	0 - 10^{9}
Maximum growth rates	v_S, v_{DP}, v_C, v_{CP}, v_{CI} hr^{-1}	0.525 1.0
Rate constant of plasmid transfer	γ cells x ml/hour	10^{-8}
Segregation rate CP-C, DP→S	τ per cell per hour	10^{-4}

REFERENCES

1. J. Monod, The growth of bacterial cultures. *Annual Review of Microbiology* **3**, 371-394 (1949).
2. F. M. Stewart, B. R. Levin, Resource partitioning and the outcome of interspecific competition: a model and some general considerations. *American Naturalist* **107**, 171-198 (1973).
3. B. R. Levin, F. M. Stewart, L. Chao, Resource - limited growth, competition , and predation: a model and experimental studies with bacteria and bacteriophage. *American Naturalist* **977**, 3-24 (1977).
4. J. Gurney, M. Pleska, B. R. Levin, Why put up with immunity when there is resistance: an excursion into the population and evolutionary dynamics of restriction-modification and CRISPR-Cas. *Philos Trans R Soc Lond B Biol Sci* **374**, 20180096 (2019).
5. W. N. Chaudhry *et al.*, Leaky Resistance and the conditions for the existience of lytic bacteriophage. *PLoS Biology* **16:e2005971** (2018).
6. F. Stewart, B. Levin, The population biology of bacterial viruses: why be temperate. *Theoretical population biology* **26**, 93-117 (1984).
7. F. M. Stewart, B. R. Levin, The Population Biology of Bacterial Plasmids: A PRIORI Conditions for the Existence of Conjugationally Transmitted Factors. *Genetics* **87**, 209-228 (1977).
8. R. Levins, Strategy of Model Building in Population Biology. *American Scientist* **54**, 421-431 (1966).
